# Trans-Species Fecal Transplant Revealed the Role of the Gut Microbiome as a Contributor to Energy Metabolism and Development of Skeletal Muscle

**DOI:** 10.3390/metabo12080769

**Published:** 2022-08-21

**Authors:** Liyuan Cai, Min Li, Shuyi Zhou, Xiaoyan Zhu, Xianghua Zhang, Qingbiao Xu

**Affiliations:** College of Animal Sciences and Technology, Huazhong Agricultural University, Wuhan 430070, China

**Keywords:** pigs, mice, gut microbiome, fecal microbiota transplantation, skeletal muscle development

## Abstract

The aim of this study was to investigate the influence of the exogenous gut microbiome at early life stages on the development of mice skeletal muscle in adulthood. First, the characteristics of skeletal muscle and the gut microbiota composition of the gut microbiota donors—Erhualian (EH) pigs (a native Chinese breed)—were studied. EH pigs had significantly higher fiber densities and thinner fiber diameters than Duroc × Landrace × Yorkshire crossed (DLY) pigs (*p* < 0.05). The expression levels of genes related to oxidized muscle fibers, mitochondrial function, and glucose metabolism in the skeletal muscle of EH pigs were significantly higher than those in DLY pigs (*p* < 0.05). Moreover, the abundances of 8 gut microbial phyla and 35 genera correlated with the skeletal muscle fiber diameters and densities exhibited significant differences (*p* < 0.05) between EH and DLY pigs. Subsequently, newborn mice were treated with saline (CG) and fecal microbiota suspensions collected from EH pigs (AG), respectively, for 15 days, starting from the day of birth. In adulthood (60 days), the relative abundances of *Parabacteroides*, *Sutterella*, and *Dehalobacterium* were significantly higher in the feces of the AG mice than those of the CG mice. The microbes contribute to improved functions related to lipid and carbohydrate metabolism. The weight, density, and gene expression related to the oxidized muscle fibers, mitochondrial function, and glucose metabolism of the AG group were significantly higher than those of the CG group (*p* < 0.05), whereas the fiber diameters in the skeletal muscle of the AG mice were significantly lower (*p* < 0.05) than those of the CG mice. These results suggested that intervention with exogenous microbiota at early stages of life can affect the fiber size and energy metabolism of their skeletal muscle.

## 1. Introduction

Gut microbiota is regarded as a “microbial organ” for animals that provides the host with certain functions that are not encoded in its genome but are vital to the host’s physiological processes, such as maintenance of the host’s health states, nutrient utilization, energy metabolism, and immune regulation [1]. When animals are born, their gut microbiota start to colonize; however, the gut microbiota of newborn animals is in an unstable state and is susceptible to external environmental factors [1]. During growth of the newborn, its gut microbiota gradually reaches a stable state, and tends to be stable when animals reach adulthood [2]. The colonization of the gut microbiota is regulated by multiple factors, such as the host’s genetic background, physiological stage, metabolism characteristics, microbe–microbe interactions, and host–microbe interplay [3,4]. Previous reports focused on the influence of exogenous gut microbiota on the gut microbiota composition and function of germ-free mice, and on the resulting changes in organ and tissue phenotypes [5,6,7]. In view of the colonization characteristics of the gut microbiota of animals, intervention with the microbiota at early life stages is an effective method for regulating its composition and characteristics. However, few studies have paid attention to the early colonization of gut microbiota.

The skeletal muscle is one of the dominant metabolic organs in the body, but to date, few studies have investigated how the gut microbiome affects skeletal muscle metabolism. With the increasing investigation of the gut microbiome–muscle axis, the role of the gut microbiota in the metabolism and development of skeletal muscle has been confirmed. It was found that some microbial taxa play vital roles in influencing the function of skeletal muscle by producing metabolic mediators that could affect host physiology after intestinal mucosa absorption [8]. However, the causal relationship between different gut microbiota and skeletal muscle characteristics has not been established in previous studies.

Erhualian (EH) pigs, a well-known indigenous pig breed in China, display specific skeletal muscle characteristics, including thinner muscle fiber diameters, higher muscle fiber densities, and a higher proportion of oxidized fibers in skeletal muscle [9]. A previous study showed that some skeletal muscle phenotypes of a donor could be transferred to a recipient through fecal microbiota transplantation (FMT) [6]. Therefore, the prominent characteristics of the skeletal muscle of EH pigs make it preferable as a fecal microbiota donor for investigating the relationships between gut microbiota and skeletal muscle characteristics. The objective of this study was to investigate whether the induction of exogenous gut microbiota derived from EH pigs can alter the fiber size and energy metabolism of their skeletal muscle by altering the gut microbiota composition of mice. This study provides a reference for the regulation of the skeletal muscle characteristics of animals by interfering with the composition of the gut microbiota at the early stages of animal life.

## 2. Materials and Methods

### 2.1. Animal Treatments

This study was conducted according to the guidelines of the University of Huazhong Agricultural Institutional Animal Care and Use Committee (Approval number: HZAUMO-2019-062). Twenty-week-old male EH pigs (*n* = 6) and male Duroc × Landrace × Yorkshire crossed (DLY) pigs (*n* = 6) were used as fecal microbiota donors in this study. EH pigs were provided by a reservation farm located in Yichang (Hubei, Wuhan, China). DLY pigs were provided by the farm of Huazhong Agricultural University. The pigs were fed a corn–soybean diet twice a day (at 08:00 and 17:00) according to the NRC (2012) recommendations for 25–50 kg pigs and had free access to water. The pigs met the donor screening criteria for fecal microbiota donors for FMT. The pigs were fed no antibiotics or probiotics for 8 weeks prior to fecal collection [10,11]. After 12 h of fasting, fresh feces of all the pigs were collected in the morning, and the fecal samples from each breed were mixed and then used to prepare the fecal microbial inocula for FMT. The remaining fecal samples were stored at −80 °C until DNA extraction. Then, all the pigs were sacrificed under the guidelines of the Laboratory Animal Ethics Committee of Huazhong Agricultural University. The *longissimus dorsi* samples of each pig were also collected and cut into 3 cm × 2 cm slices following the direction of the muscle fibers in the center of the muscle, and then fixed with 4% paraformaldehyde overnight.

### 2.2. Preparation and Transplantation of Fecal Microbiota

The fecal samples were diluted with sterile saline (1:5, *v*/*v*), homogenized in a standard blender in a biological cabinet filled with nitrogen, and then passed through a sterilized tea strainer to remove large particles [10,11]. The fecal suspension was then filtered three times through 0.25 mm stainless steel sieves to eliminate the small particles. The live microbiota in the suspension were counted by optical microscopy combined with methylene blue staining. Twenty-four newborn specific pathogen-free C57BL/6J mice of both sexes were provided by the experimental animal center of Huazhong Agricultural University. The newborn mice were divided into three groups and treated with fecal microbial suspensions originating from EH pigs (AG group, *n* = 12) or sterile saline (CG group, *n* = 12). The concentration of the fecal microbial suspension used for FMT was 1.0 × 10^8^ CFU/mL. The volume of sterile saline or microbial suspension was gradually increased from 0.20 to 0.50 mL for the period from newborn to 7 days of age and for the period from 7 to 15 days of age, respectively, and this volume was administered once daily by a feeding needle device through oral gavage. The mice were weaned at 21 days of age and then given ad libitum access to feed and water.

### 2.3. Sample Collection

At the end of the study (day 90), freshly excreted mouse feces were collected from each group and immediately frozen in liquid nitrogen and stored at −80 °C. Then, all the mice were sacrificed according to the guidelines of the Laboratory Animal Ethics Committee of Huazhong Agricultural University. Blood samples were collected after eyeball enucleation and placed at room temperature (25 °C) for 30 min, were then centrifuged at 3000 r/min for 10 min to obtain serum, and were stored at −80 °C until analysis. Skeletal muscle samples from the legs of the mice, including the *quadriceps femoris*, *anterior tibialis* muscle, *gastrocnemius*, *soleus*, and *extensor digitorum longus*, were collected and weighed. The muscles were then cut into 3.0 cm × 2.0 cm slices following the direction of the muscle fibers in the center of the muscle and fixed with 4% paraformaldehyde overnight.

### 2.4. 16S rRNA Sequencing and Analysis

Microbial DNA was extracted and purified from approximately 0.25 g of the feces from pigs and mice using the QIAamp Stool DNA Mini Kit (QIAGEN, Dusseldorf, Germany) according to the manufacturer’s instructions. The concentration and purity of DNA were measured using a NanoDrop ND-1000 Spectrophotometer (Thermo Fisher Scientific, Waltham, MA, USA). The quality of the genomic DNA was determined by electrophoresis on 1% agarose gels. The V3-V4 variable regions of the bacterial 16S rRNA gene were amplified using the primers 341F (5′-ACTCCTACGGGAGGCAGCAG-3′) and 806R (5′-GGACTACHVGGGTWTCTAAT-3′). Their primers were tagged with Illumina adapter, pad, and linker sequences. PCR enrichment was performed in a 50 μL reaction containing 30 ng of template fusion PCR primer and PCR master mix. The PCR cycling conditions were used as follows: 94 °C for 3 min, 30 cycles of 94 °C for 30 s, 50 °C for 45 s, 72 °C for 45 s, and a final extension at 72 °C for 10 min. The PCR products were purified with AMPure XP beads and eluted in elution buffer. The quality of the libraries was verified using an Agilent 2011 bioanalyzer (Agilent, Santa Clara, CA, USA). The validated libraries were used for sequencing on the Illumina MiSeq platform (BGI, Shenzhen, China) following the standard pipelines of Illumina to generate 2 × 300 bp paired-end reads. The resulting sequence reads were filtered to remove adaptors and low-quality and ambiguous bases to obtain the tags using FLASH (v1.2.11) [12]. The chimeric sequences were detected and filtered using UCHIME (v4.2.40) [13], and operational taxonomy units (OTUs) were generated by clustering non-chimeric sequences with a cutoff value of 97% using UPARSE software (v7.0.1090) [14]. USEARCH_global was used to align all the reads back to the centroids of the OTUs to estimate the relative abundances [15]. The representative sequence of each OTU was taxonomically classified using the RDP Classifier (v2.2) by QIIME (v1.9.0) [16]. Each sequence was annotated for species classification and compared with the Greenene Database, and the comparison threshold was 0.80. Alpha diversity indices (Chao1, Shannon, Simpson, and Good’s coverage indices) and beta diversity values based on weighted UniFrac distances were calculated using QIIME (v1.9.0). Principal component analysis (PCA) plots were produced based on weighted UniFrac metrics using the factoextra package (v1.0.7) in R software. A permutational multivariate analysis of variance (PERMANOVA) based on weighted UniFrac matrices was performed with 1000 permutations to test the differences in the fecal microbiota among groups using a vegan package. The microbial 16S rRNA genes were subjected to Phylogenetic Investigation of Communities by Reconstruction of Unobserved States (PICRUSt2) against the Kyoto Encyclopedia of Genes and Genomes (KEGG) database for predicting KEGG Orthology (KO) [17]. The relative abundance of the metabolic pathways was then calculated.

### 2.5. Histological Assessment of the Skeletal Muscle Fiber Morphology

The *longissimus dorsi* (of pigs) and the quadriceps muscle (of mice) were embedded in paraffin. Each embedded muscle was sectioned into 4.0-µm-thick transversal sections and stained with hematoxylin-eosin (HE). Ten muscle fibers of each sample were observed using an Olympus BX53 microscope (Olympus Corporation, Tokyo, Japan). ImageJ software (v1.8.0) was used for the measurement of the skeletal muscle fiber diameters and for counting the muscle fiber numbers.

### 2.6. Skeletal Muscle Gene Expression Analysis by RT–PCR

Total RNA was extracted from the *longissimus dorsi* of the pigs and QF of the mice using TRIzol^®^ Reagent (Life Technologies, Carlsbad, CA, USA) and then reverse-transcribed using a Revert Aid First Strand cDNA Synthesis kit (Thermo Fisher Scientific, Waltham, MA, USA) by following the manufacturer’s instructions. Primers were designed using Primer 5.0 software (Primers Co. Ltd., Foster, CA, USA) or based on the information provided in Table 1, as previously reported [7,9,18,19]. The primers were obtained from Sangon Biotech Co., Ltd. (Shanghai, China). RT–PCR was conducted using a SYBR RT–PCR Kit (Bio–Rad, Hercules, CA, USA) in conjunction with an ABI QuantStudio TM6 Flex real-time fluorescent quantitative PCR system (Life Technologies, Carlsbad, CA, USA). To ensure the accuracy of the test results, each sample was analyzed in triplicate. The gene expressions were quantified using the 2^−ΔΔCt^ method [20].

### 2.7. Glycogen Measurements in Skeletal Muscle

The glycogen content in the skeletal muscle samples was measured using a glycogen assay kit (Bioengineering Institute, Nanjing, Jiancheng, China) by following the manufacturer’s instructions.

### 2.8. Statistical Analysis

In the present study, only the microbial taxa or annotated functions at different levels with a relative abundance higher than 0.05% in at least 50% of individuals within each experimental group were considered as being detected and used for further analysis. The bacterial taxa composition and alpha diversity indices were analyzed using the Kruskal–Wallis test, and the Dunn test was applied for multiple pairwise comparisons between groups. One-way analysis of variance (ANOVA) and Duncan’s method were used for multiple comparisons of the skeletal phenotypes, gene expression, and immune parameters between the CG and AG groups. A two-tailed Student’s *t* test was performed using GraphPad Prism 8 statistical software (GraphPad Software Inc., San Diego, CA, USA). A value of *p* < 0.05 was considered statistical significance. The values are expressed as the means ± SEM. The significantly different bacterial genera and KEGG pathways were used in the correlation analysis based on Spearman’s rank correlation coefficients and conducted with the Hmisc package. The cutoffs for the correlation coefficients and *p*-values were determined to equal 0.50 and 0.05, respectively.

## 3. Results

### 3.1. Fiber Size and Energy Metabolism Characteristics in the Skeletal Muscle of EH Pigs

**Figure 1:**
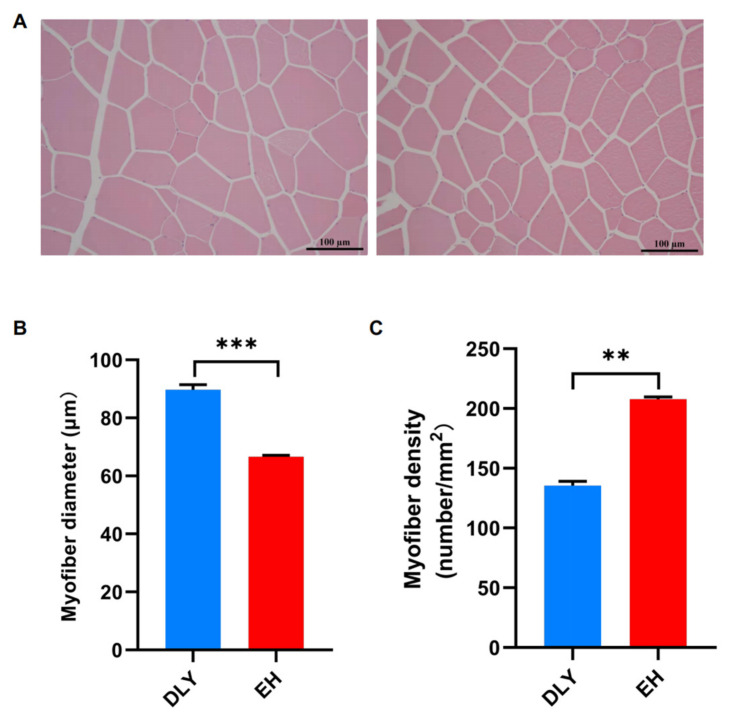
Fiber size of the *longissimus dorsi* of DLY and EH pigs. (**A**) HE staining (original magnification, ×200) showed the fiber size of the *longissimus dorsi* of DLY (left) and EH pigs (right). (**B**) Fiber diameters and (**C**) densities of the *longissimus dorsi*. The data were expressed as means ± SEM; ** *p* < 0.01 and *** *p* < 0.001.

The fiber size of the *longissimus dorsi* was observed by HE staining (Figure 1A). The skeletal muscle fibers of EH pigs were significantly thinner than those of DLY pigs (*p* < 0.05; Figure 1B); however, the skeletal muscle fiber densities of EH pigs were significantly higher than those of DLY pigs (*p* < 0.05; Figure 1C). These results suggested that the meat of native EH pigs had better taste and flavor than that of DLY pigs.

**Figure 2:**
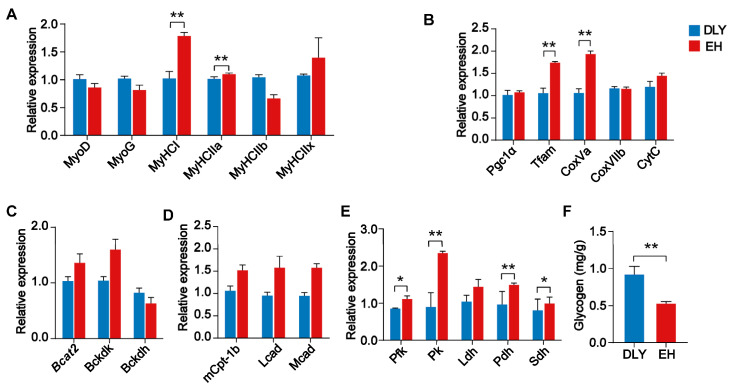
Relative mRNA expression of genes in the *longissimus dorsi* of DLY and EH pigs. (**A**) Expression of the skeletal muscle-specific transcription factors, *MyoD* and *MyoG*, which encode myosin heavy chain (*MyHC*) isoforms, namely, the *MyHC I*, *MyHC IIa*, *MyHC IIb*, and *MyHC IIx* genes. (**B**) Expression of the *PGC-1α*, *Tfam*, *CoxVa*, *CoxVIIb*, and *Cyt c* genes related to mitochondrial function. (**C**) Expression of the *Bcat2* and *Bckdk* genes involved in branched-chain amino acid catabolism. (**D**) Expression of the *Pfk*, *Pk*, *Ldh*, *Pdh*, and *Sdh* genes, which are involved in glucose metabolism. (**E**) Expression of the *mCpt-1b*, *Lcad*, and *Mcad* genes involved in the fatty acid oxidation pathway. (**F**) Glycogen concentration. The data were expressed as the means ± SEM; * *p* < 0.05, ** *p* < 0.01.

The expression in the *longissimus* *dorsi* of genes related to skeletal muscle growth, differentiation, and fiber types was evaluated. The *longissimus* *dorsi* of EH pigs exhibited significantly higher expression of the *MyHCI* and *MyHCIIa* genes (*p* < 0.01) (Figure 2A). To examine the oxidative metabolic capacity of skeletal muscle, the expression of genes related to mitochondrial function was investigated. The *longissimus dorsi* of EH pigs exhibited significantly higher expression of the *Tfam* and *CoxVa* genes (*p* < 0.01) (Figure 2B). Furthermore, the expression of genes related to lipid metabolism and glucose metabolism was investigated, and the results showed that the *longissimus dorsi* of EH pigs exhibited significantly higher expression of the *Pfk*, *Pk*, and *Pdh* genes (*p* < 0.05) (Figure 2C–E). In addition, decreased glycogen accumulation was observed in the *longissimus dorsi* of EH pigs compared with that of DLY pigs (*p* < 0.01; Figure 2F).

### 3.2. Composition and Function of the Gut Microbiota of EH Pigs

Good’s coverage index showed that the 16S rRNA gene sequences covered more than 99.50% of the microbial taxa in the feces of both the EH and DLY pigs (Table 2). The dominant microbial phyla in the feces of the EH and DLY pig breeds included *Bacteroidetes* (54.89 ± 2.39%), *Firmicutes* (36.07 ± 1.62%), and *Proteobacteria* (4.63 ± 0.78%). The dominant microbial genera were *Prevotella* (41.00 ± 3.74%), *Phascolarctobacterium* (3.39 ± 0.39%), and *Lachnospira* (3.09 ± 0.32%). A comparison of the alpha diversity indices showed that the EH pigs exhibited significantly higher Chao1 and Shannon indices and a significantly lower Simpson index than the DLY pigs (*p* < 0.05; Table 2).

**Figure 3:**
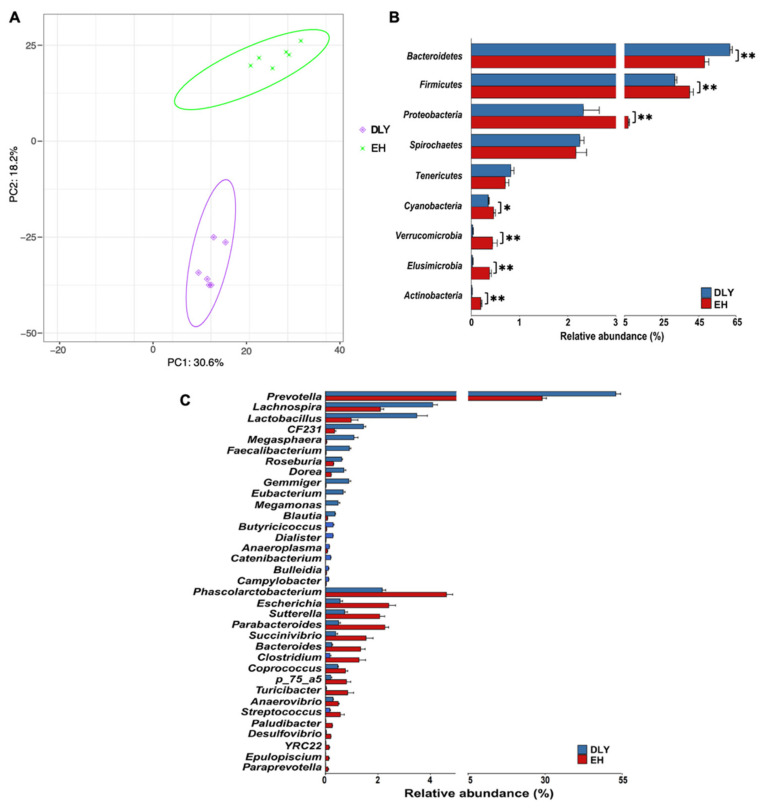
Profiles of the gut microbiota composition of DLY and EH pigs. (**A**) Profiles of the gut microbiota composition based on genera and visualized by PCA. (**B**) Gut microbiota composition at the phylum level. (**C**) Gut microbiota genera showing differential abundance between DLY and EH pigs. Data were expressed as means ± SEM; * *p* < 0.05, ** *p* < 0.01.

The PCA based on weighted UniFrac metrics showed clear separation of the fecal microbiota between EH and DLY pigs (Figure 3A). At the phylum level, the relative abundances of *Firmicutes*, *Proteobacteria*, and *Cyanobacteria* were significantly higher in the feces of EH pigs than in the feces of DLY pigs (*p* < 0.05), whereas that of *Bacteroidetes* was significantly higher in the feces of DLY pigs than that in the feces of EH pigs (*p* < 0.01; Figure 3B). At the genus level, the relative abundances of *Phascolarctobacterium*, *Escherichia*, *Sutterella*, *Parabacteroides*, and 13 other genera were significantly higher in the feces of EH pigs than those in the feces of DLY pigs (*p* < 0.05), whereas *Prevotella*, *Lachnospira*, *Lactobacillus*, *CF231*, and 14 other genera were found at significantly higher abundances in the feces of DLY pigs than those in the feces of EH pigs (*p* < 0.05; Figure 3C).

**Figure 4:**
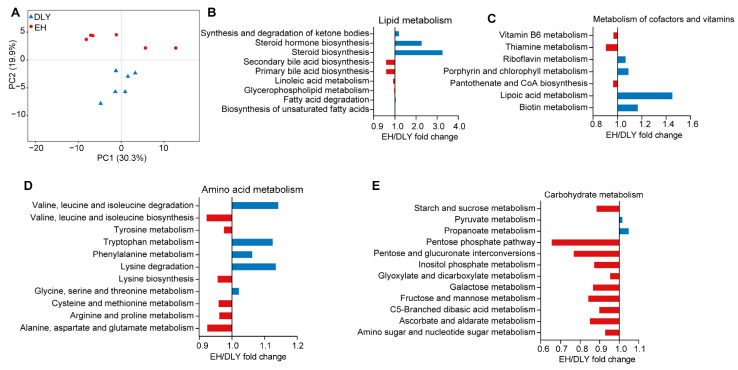
EH/DLY fold change showed differences in KEGG level-3 microbial metabolic pathways between DLY and EH pigs. (**A**) The metabolism profiles of gut microbiota of DLY and EH pigs based on KEGG level-3 “Metabolism” pathways visualized by PCA. (**B**) Lipid metabolism. (**C**) Metabolism of co-factors and vitamins. (**D**) Amino acid metabolism. (**E**) Glucose metabolism.

Based on the KEGG gut microbial function analysis, 143 endogenous third-level pathways were considered gut microbial metabolic pathways, which belonged to four KEGG level-1 categories: “metabolism” (78.70 ± 0.18%), “genetic information processing” (14.90 ± 0.15%), “cellular processes” (4.52 ± 0.04%), and “environmental information processing” (1.87 ± 0.06%). In the level-2 categories, 29 pathways were detected, and “metabolism of co-factors and vitamins” (13.92 ± 0.19%), “carbohydrate metabolism” (13.38 ± 0.56%), and “amino acid metabolism” (12.78 ± 0.18%) were the three most abundant pathways. PCA showed two separated clusters based on KEGG level-3 “Metabolism” pathway profiles of gut microbiota between the two pig groups (Figure 4A). There were 39 level-3 pathways belonging to the “Metabolism” category that were significantly differentially abundant in the gut microbiota between EH and DLY pigs, including lipid metabolism, metabolism of vitamins, amino acid, and carbohydrate pathways, which were significantly enriched in the gut microbiota of EH pigs compared to DLY pigs (*p* < 0.05; Figure 4B–E).

### 3.3. Correlations between Different Microbial Genera and Skeletal Muscle Phenotypes of Pigs

**Figure 5:**
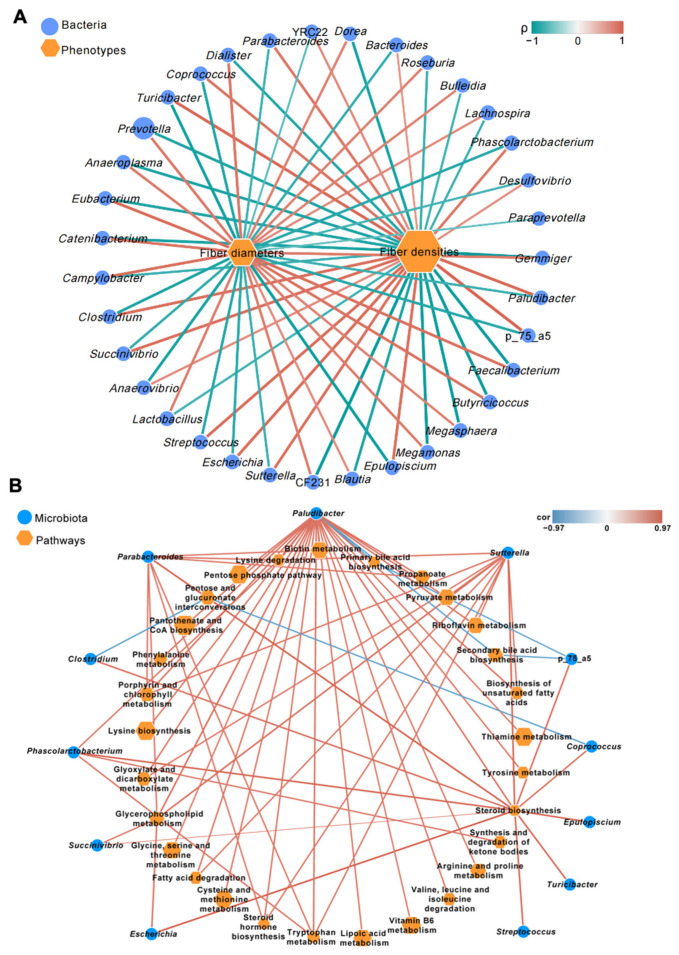
Correlation networks showed the relationships between differential microbial genera and skeletal muscle phenotypes (**A**) or microbial KEGG level-3 metabolic pathways (**B**) of pigs. (**A**) The node size and color (blue circle: differential microbial genera; orange hexagon: differential skeletal muscle phenotypes) are proportional to the mean relative abundance in the respective population. The lines’ width and color (red: positive; green: negative) are proportional to the correlation strength. Only significant correlations (R > 0.60 or <−0.60; *p* < 0.05) are displayed. (**B**) The node size and color (blue circle: differential microbial genera; orange hexagon: differential microbial metabolic pathways) are proportional to the mean relative abundance in the respective population. Only significant correlations (R > 0.75 or <−0.75; *p* < 0.05) were displayed.

The correlations of different gut microbial genera with skeletal muscle phenotypes (fiber diameter and density) were examined by Spearman’s rank correlation analysis. All 35 significantly different genera showed significant relationships with the fiber diameters and densities of skeletal muscle (coefficient R > 0.50 or <−0.50; *p* < 0.05; Figure 5A). The different microbes that were highly positively associated with fiber density and highly negatively associated with fiber diameter (coefficient ρ > 0.70 or <−0.70; *p* < 0.001) were considered phenotype-correlated microbial genera. Twelve phenotype-correlated microbial genera were detected in this study, including Succinivibrio, Parabacteroides, and Sutterella (Figure 5A).

A correlation analysis revealed 397 significant correlations (coefficient R > 0.50 or <−0.50; *p* < 0.05) between phenotype-correlated microbial genera and significantly different KEGG level-3 “Metabolism” pathways. The top nine highly positive correlations were all related to the “Steroid biosynthesis” pathway (coefficient ρ > 0.85; *p* < 0.0001; Figure 5B). Succinivibrio, Parabacteroides, and Sutterella were the gut microbes with the three highest correlations with this pathway (coefficient R > 0.93; *p* < 0.0001). The high steroid biosynthesis in the skeletal muscle indicated high intramuscular fat in the better quality meat. Therefore, these gut microbes may contribute to the high quality of the skeletal muscle.

### 3.4. Gut Microbiota Transplantation Altered Fiber Size and Energy Metabolism in Mice Skeletal Muscle

**Figure 6:**
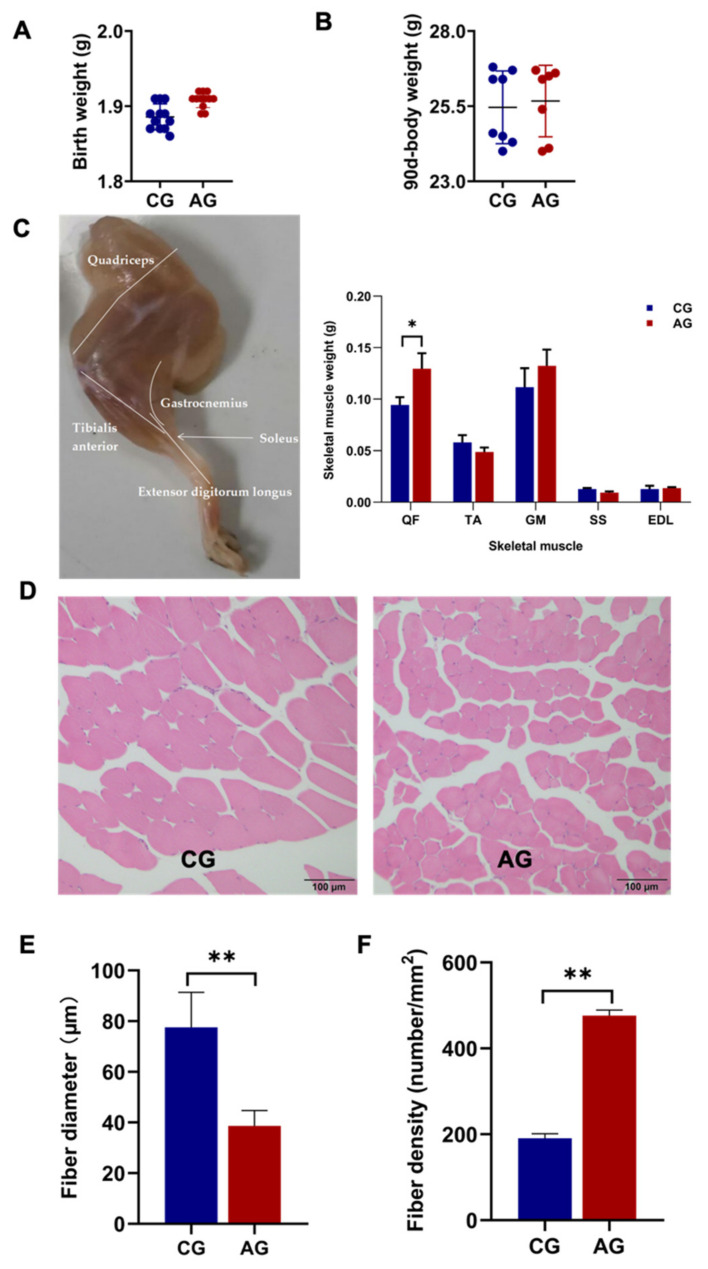
Body weight and skeletal muscle histological characteristics of mice. (**A**) Birth weight. (**B**) 90-day body weight. (**C**) The weights of quadriceps femoris (QF), tibial anterior muscle (TA), gastrocnemius (GM), soleus (SS), and extensor digitorum longus (EDL). (**D**) HE staining (original magnification, ×200) showed the fiber size of quadriceps femoris. (**E**) The fiber diameters and (**F**) densities in quadriceps femoris. The data were expressed as the means ± SEM; * *p* < 0.05, ** *p* < 0.01.

The results showed that the body weights of the mice at birth and day 90 were not significantly different between the CG and AG group (Figure 6A,B). The weight of the QF, a fast glycolytic muscle, was significantly increased in the AG group compared with the CG group (*p* < 0.01; Figure 6C). A histological examination of the QF revealed significantly thinner fiber diameters and higher fiber densities (*p* < 0.01, Figure 6D–F) in the AG group than that in the CG group.

**Figure 7:**
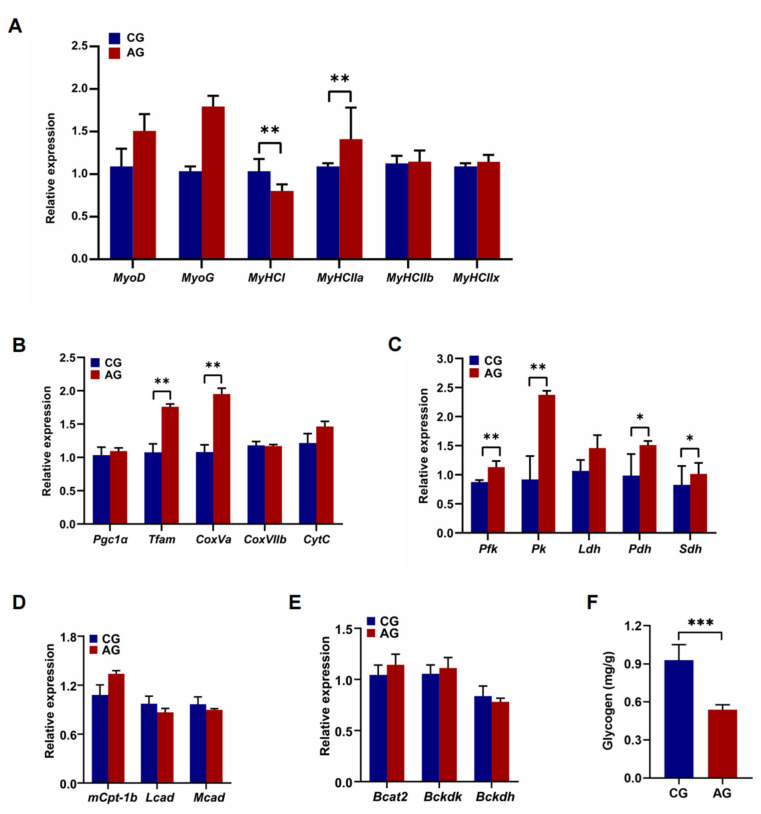
Gene expression in quadriceps femoris (QF) of mice. (**A**) The expression of the skeletal muscle-specific transcription factors, *MyoD* and *MyoG* genes, which encode myosin heavy chain (*MyHC*) isoforms, namely, the *MyHC I*, *MyHC IIa*, *MyHC IIb*, and *MyHC IIx* genes in the QF of CG and AG mice. (**B**) The expression of the *PGC-1α*, *Tfam*, *CoxVa*, *CoxVIIb*, and *Cyt c* genes related to mitochondrial function in the QF of CG and AG mice. (**C**) The expression of the *Pfk*, *Pk*, *Ldh*, *Pdh*, and *Sdh* genes, which are involved in glucose metabolism in the QF of CG and AG mice. (**D**) The expression of the *mCpt-1b*, *Lcad*, and *Mcad* genes involved in the fatty acid oxidation pathway in the QF of CG and AG mice. (**E**) The expression of the *Bcat2* and *Bckdk* genes involved in branched-chain amino acid catabolism in the QF of CG and AG mice. (**F**) The concentration of glycogen in the QF of CG and AG mice. Data are expressed as mean ± SEM. * *p* < 0.05, ** *p* < 0.01, and *** *p* < 0.001.

The gene expression in QF related to skeletal muscle growth, differentiation, and fiber types, including MyoD, MyoG, MyHCI, MyHC IIa, MyHC IIb, and MyHC IIx, was examined. The expression of the MyHC I and MyHC IIa genes was significantly increased in the AG mice compared to the CG mice (*p* < 0.05). There were no significant differences in the expression of MyoD, MyoG, MyHC IIb, and MyHC IIx genes in QF between the CG and AG group (Figure 7A). To examine the oxidative metabolic capacity of skeletal muscle, the gene expression related to mitochondrial function, glucose metabolism, lipid metabolism, and branched-chain amino acid metabolism was investigated. The expression levels of Tfam, CoxVa, Pfk, Pk, Pdh, and Sdh were significantly higher in the QF of the AG group than that in CG group (*p* < 0.05; Figure 7B,C). There was no significant difference in the expression of the Pgc-1α, CoxVIIb, CytC, Ldh, mCpt-1b, Lcad, Mcad, Bcat2, Bkcdk, and Bckdh genes in QF between the AG and CG group (Figure 7B–E). In addition, decreased glycogen accumulation in the QF of the AG group was observed compared to that of the CG group (*p* < 0.001; Figure 7F).

### 3.5. Gut Microbiota Composition and Functional Profiles of Mice

**Figure 8:**
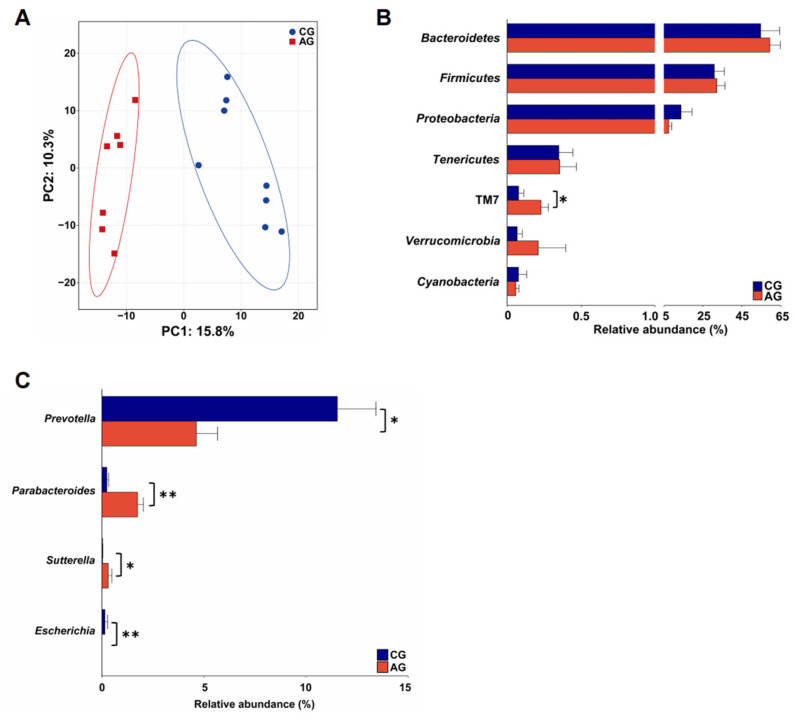
Profiles of gut microbiota composition in mice. (**A**) Gut microbiota compositional profiles of CG and AG mice based on genera visualized by PCA. (**B**) Gut microbiota composition at the phylum level of CG and AG mice. All the microbial phyla are displayed. (**C**) Differential gut microbiota genera between the CG and AG mice. Data are expressed as mean ± SEM. * *p* < 0.05 and ** *p* < 0.01.

According to Good’s coverage index, the number of sequences from the 16S rRNA gene-sequencing datasets were sufficient to cover more than 99% of the microbial taxa (Table 2). The dominant microbial phyla in the feces of mice included *Bacteroidetes* (53.83 ± 4.34%), *Firmicutes* (35.20 ± 3.07%), and *Proteobacteria* (10.14 ± 2.31%). The dominant microbial genera were *Helicobacter* (7.64 ± 2.27%), *Prevotella* (7.18 ± 1.13%), and *Bacteroides* (6.03 ± 1.22%). Compared with the CG group, the Chao1 and Shannon indices were significantly higher in the AG group (*p* < 0.05), while the Simpson index was significantly lower in the AG group than that in the CG group (*p* < 0.05; Table 2). The PCA showed a distinct separation of the fecal microbiota between pigs and mice (Figure 8A). Only seven of the same microbiota genera were found in the feces of both pigs and mice. The relative abundances of *Bacteroides, Clostridium*, *Parabacteroides,* and *Sutterella* were significantly higher, while those of *Anaeroplasma*, *Butyricicoccus,* and *Roseburia* were significantly lower in the feces of EH pigs than in those of DLY pigs. The relative abundance of the phylum, TM7, was significantly higher in the AG group than that in the CG group (*p* < 0.05; Figure 8B). At the genus level, *Parabacteroides*, *Sutterella*, and *Dehalobacterium* were enriched in the feces of AG mice compared with CG mice, while *Prevotella*, *Anaerostipes*, and *Escherichia* were enriched in the feces of the CG group (*p* < 0.05; Figure 8C).

**Figure 9:**
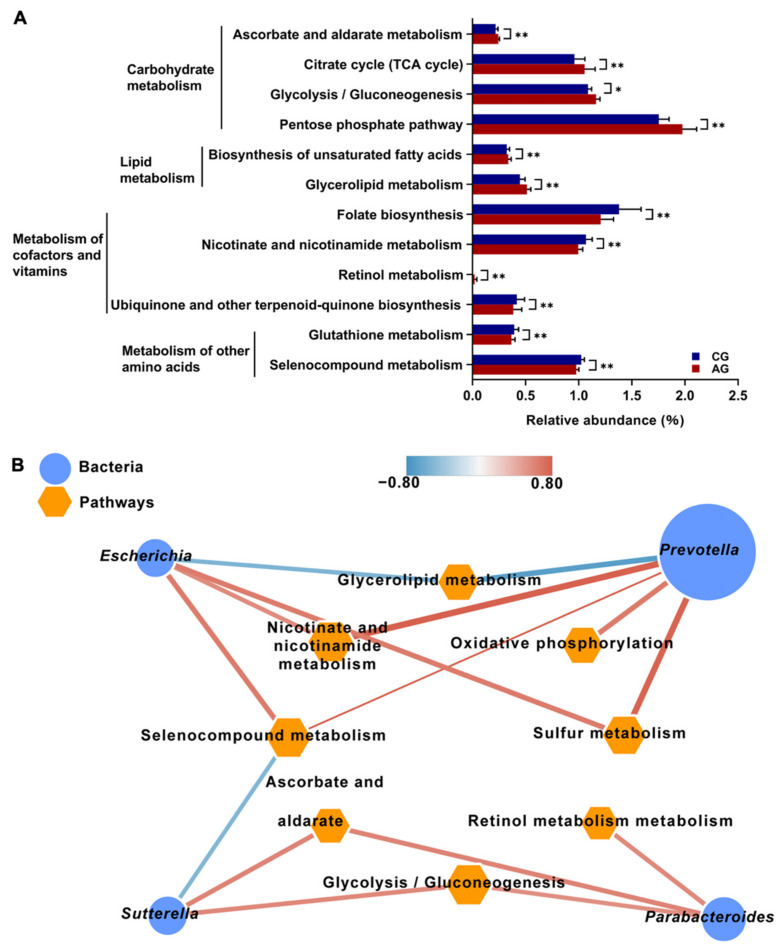
Functions of the gut microbiota of mice. (**A**) Relative abundance of KEGG level-3 metabolic pathways showing differences between the CG and AG groups. (**B**) Correlation networks showing the relationships between differential microbial species and the differential microbial KEGG level-3 metabolic pathways. The node size and color (red circle: differential microbial genera; orange hexagon: differential microbial metabolic pathways) are proportional to the mean relative abundance in the respective population. The width and color of the lines (red: positive; blue: negative) are proportional to the correlation strength. Only significant correlations (R > 0.60 or <−0.60; *p* < 0.05) are displayed. Data are expressed as mean ± SEM. * *p* < 0.05 and ** *p* < 0.01.

A total of 144 metabolic pathways were identified at KEGG level 3. These predicted pathways belonged to 6 categories at KEGG level 1, including “Metabolism” (78.07 ± 0.44%), “Cellular Processes” (13.98 ± 0.09%), “Genetic Information Processing” (5.67 ± 0.28%), and “Environmental Information Processing” (2.26 ± 0.08%). At KEGG level 2, 28 pathways were detected, with “carbohydrate metabolism” (14.75 ± 0.09%), “metabolism of co-factors and vitamins” (12.61 ± 0.05%), and “xenobiotic biodegradation and metabolism” (12.32 ± 0.20%) being the top three abundant pathways. Among all level-3 pathways, 107 metabolic pathways were identified, and a PCA did not show clear separation of the KEGG level-3 metabolism pathways, but a comparison of the relative abundance of KEGG level-3 pathways identified a total of 12 metabolic pathways that showed significant differences between the CG and AG groups. Four “carbohydrate metabolism” pathways, two “lipid metabolism” pathways, and one “metabolism of co-factors and vitamins” pathway were significantly enriched in the AG group compared with the CG group (*p* < 0.05; Figure 9A). The other five different pathways were enriched in the CG group compared with the AG group (*p* < 0.05; Figure 9A). A correlation analysis using Spearman’s rank correlation coefficients revealed various significant correlations between significantly different microbial genera and significantly different metabolism pathways. A total of 25 significant correlations were identified (coefficient ρ > 0.50 or <−0.50; *p* < 0.05). The top three correlations were related to *Prevotella*: “ko00760: nicotinate and nicotinamide metabolism” (ρ = 0.83, *p* < 0.05), “ko00450: seleno compound metabolism” (ρ = 0.79; *p* < 0.05), and “ko00790: folate biosynthesis” (ρ = 0.77; *p* < 0.05). Moreover, *Parabacteroides* was significantly correlated with the “carbohydrate metabolism” and “metabolism of cof-actors and vitamin metabolism” pathways, including “ko00010: glycolysis/gluconeogenesis” (ρ = 0.69; *p* < 0.05) and “ko00830: retinol metabolism” (ρ = 0.65; *p* < 0.05).

## 4. Discussion

As the gut microbiota–skeletal muscle axis has been increasingly investigated, various studies have focused on the role of gut microbes in skeletal muscle metabolism [8]. Experimental mice exhibited significantly lower expression of the phosphoric acid, fructose kinase, pyruvate kinase, pyruvate dehydrogenase, and lactate dehydrogenase genes, which are related to the metabolism of glucose and fatty acids in the tibialis anterior muscle, soleus, and extensor digitorum longus, and exhibited significantly increased glycogen accumulation in the quadriceps muscle compared with specific-pathogen free and germ-free mice [7]. The transfer of the pig gut microbiota to germ-free mice induces changes in the skeletal muscle fiber diameter, density, and cross-sectional area in mice [6]. These results suggested that the gut microbiota can affect the characteristics of skeletal muscle. These studies have established the correlational foundation for the present study. Mice are a suitable model for studying the interactions between the gut microbiota and other metabolisms of the host [21,22,23]. The FMT approach was applied in this study to explore whether intervention with exogenous gut microbiota in mice at the early life stages can influence the composition of the gut microbiome in adult mice and thus cause changes in their skeletal muscle phenotype and energy metabolism.

Despite the hereditary factors, the difference of mycofiber diameter and mycofiber density between DLY and EH pigs may have a connection with the gut microbiome. To verify this hypothesis, the skeletal muscle characteristics and gut microbial composition and functional characteristics obtained after exposure to exogenous gut microbiota from EH pig donors were explored in this study. Thinner fiber diameters and higher fiber densities were found in the *longissimus dorsi* of EH pigs compared with that of DLY pigs. Moreover, the glucose metabolism in the skeletal muscle of EH pigs was also significantly higher than that in DLY pigs. These results could serve as evidence of the unique skeletal muscle characteristics of EH pigs. The gut microbiota analysis showed that the relative abundance of *Firmicutes* was significantly higher and that of *Bacteroidetes* was significantly lower in the feces of EH pigs than those of DLY pigs. A higher ratio of *Firmicutes*/*Bacteroides* in the gut microbiota has been correlated with a higher intensity of energy absorption in the animal body and an obese phenotype [5,24]. Moreover, *Proteobacteria* is correlated with energy accumulation [25] and was found at a higher relative abundance in the feces of EH pigs than those of DLY pigs. In addition, the present study revealed that the relative abundance of *Succinivibrio* was significantly higher in the feces of EH than those of DLY pigs. The *Succinivibrio* genus can produce acetic, propionate, and succinate acids and thus affects the host’s energy metabolism [26,27]. These results revealed that the higher abundance of gut microbiota related to “enhanced energy metabolism” in EH pigs might contribute to the better skeletal muscle energy metabolism of these pigs. In addition, a correlation analysis revealed that several gut microbiota genera of EH pigs were more highly correlated with skeletal muscle phenotypes. Therefore, the gut microbiota derived from the feces of EH pigs were selected as exogenous gut microbiota for intervention with the gut microbiota in mice at early life stages.

In the present study, the QF weight of mice was increased by FMT, and similar results were obtained in a previous study after FMT to germ-free mice [7]. Additionally, the recipient mice had thinner muscle fibers in the QF than the control mice. Consistent with our results, a previous study showed that the skeletal muscle fiber diameters of germ-free mice were affected by the fecal microbiota of pigs [6]. In this study, the expression levels of the *Tfam* and *CoxVa* genes, which are related to mitochondrial function, were significantly increased in the QF of mice that were administered the exogenous gut microbiota. These results indicated that the gut microbiota exerted an appreciable effect on skeletal muscle energy metabolism. By determining the gene expressions in skeletal muscle that are related to energy metabolism, including carbohydrate metabolism, fat metabolism, and branch-chain amino acid metabolism, a significant increase was observed in the expression of the *Pfk*, *Pk*, *Pdh*, and *Sdh* genes in the skeletal muscle of mice that were administered the exogenous gut microbiota. These results suggested that the gut microbiota significantly improved some critical energy metabolism activities in the skeletal muscle of the recipient mice. Moreover, the reduction in glycogen accumulation in the QF of the recipient mice implies the potentially enhanced utilization of glucose by the QF of recipient mice. Consistent with our findings, previous studies have also suggested that the gut microbiota plays an essential role in skeletal muscle energy metabolism, which could significantly affect the expression of the *Ldh*, *Pk*, and *Sdh* genes and glycogen accumulation in the skeletal muscle of mice [7].

The results from the present study showed that the relative abundance of only a few microbial genera, such as *Bacteroides*, *Clostridium*, and *Parabacteroides*, exhibited a similar trend in the feces of pigs and mice. This result is consistent with previous studies that showed that transplantation of the fecal microbiota from pigs to mice could affect the gut microbiota composition in the recipients [28]. However, these data are inconsistent with those obtained in previous studies, which reported similar gut microbiota compositions between germ-free recipient mice and pig donors [5,6]. These results suggested that the gut microbiota composition of conventional mice was not affected by a single factor (fecal microbiota of pigs). The host and other environmental factors could also influence the gut microbial community [29,30]. Moreover, *Parabacteroides* was the microbial genus that showed the greatest difference in abundance between the AG and CG groups in this study. *Parabacteroides* can produce acetate, propionate, and succinate [31]. Acetate and propionate play an important role in skeletal metabolism by promoting insulin sensitivity, mitochondrial biogenesis, and energy metabolism [8]. Succinate is an important intermediate in the tricarboxylic acid cycle and a central player in mitochondrial metabolism for ATP generation, which induces skeletal muscle fiber remodeling via SUCNR1 signaling and changes its energy metabolism [32]. Therefore, the observed changes in the fiber size and energy metabolism could partly be attributed to the increased relative abundance of *Parabacteroides* in the gut of the recipient mice. The correlation analysis revealed that *Parabacteroides* was highly correlated with “glycolysis/gluconeogenesis” and “retinol metabolism”. The detected increase in *Parabacteroides* in the gut of the recipient mice may produce more metabolites related to the growth, development, and metabolism of skeletal muscle by these two pathways. Therefore, significant differences in the skeletal muscle fiber size and energy metabolism were found between the AG and CG groups.

## 5. Conclusions

As gut microbiota donors, EH pigs have fine fiber and high glucose metabolism levels in their skeletal muscle. The gut microbiota of this breed of pigs also exhibits unique compositions and functional characteristics, and these pigs are thus suitable gut microbiota donors for studying the relationships between gut microbiota and skeletal muscle energy metabolism. Interfering with the gut microbiota of mice at an early stage of life through exposure to the gut microbiota derived from EH pigs can change the composition of the gut microbiota and skeletal muscle characteristics of the mice in adulthood.

## Figures and Tables

**Table 1:** Primer sequences used for RT-PCR.

Gene	Primer Sequence (5’ to 3’)	GenBank No.
**For pigs**		
*Bcat2*	F: GCCTGAAGGCGTACAAAGG; R: GATGCACTCCAGCAACTCG	
*Bckdh*	F: CCAGATGCCCGTCCACTAC; R: CCCCCTCTCCGAAGTAACAG	
*Bckdk*	F: TCCGACCATGATGCTCTATTC; R: GAAGTCCTT GATGCGGTG G	
*CoxVa*	F: CCGCCGCTATCCAGTCAATTCG; R: ATTTCCTTATGAGGTCCTGCTTTGTCC	XM_005666147.2
*C* *o* *XVIIb*	F: GGCAAGGCAGAACCACCAGAAG; R: TGGGTACACAGTGCTTTTACTTGGC	XM_003135201.3
*mCpt-1b*	F: CACGGCAACTGCTACAACA; R: CCAGGACGAACTCCCAGA	
*Cyt* *C*	F: AACTGGTCAAGCACCTGGTTATAGC; R:ACCAGACGGAGAATCAATGAATGAGTC	XM_003127002.4
*GAPDH*	F: GTCGGAGTGAACGGATTTGG; R: CAATGTCCACTTTGCCAGAGTTAA	NM_001206359.1
*L* *cad*	F: TGACTGGATTCTCAATGGAAGCAAGG; R: CACTCGCTGGCAACCGTACATC	NM_213897.1
*Ldh*	F: TCTTGACCTATGTGGCTTGG; R: AGCACTGTCCACCA CCTGTT	XM_013994501.2
*Mcad*	F: GGCGAGTACCCTGTCCCACTAC; R: GCCTGCTCCTGGTTCTGTTACAC	NM_214039.1
*MyHCI*	F: CAAGGCAGAGATGGAGCGGAAG; R: CTCATTGCGGCTGCGTGTCT	NM_213855
*MyHCIIa*	F: GGAAGCTCGCAACGCAGAAGA; R: TCATCCAGACGGTGCTGTAGGT	NM_214136
*MyHCIIb*	F: CGGAAGAGGCGGAGGAACAATC; R: TGACCTGGGACTCGGCAATGT	NM_001104951
*MyHCIIx*	F: GTACAATGCGGTGGGTGCTCTG; R: GCTGCTGGTTGATGCGAGTGA	NM_001123141
*MyoD*	F: CTGCTACGACGGCACCTATT; R: CACGATGCTGGACAGACAG	NM001002824
*MyoG*	F: AGTGGAGGATGTGGCTGTG; R: AGAAGTGGTGCCGTCTGTG	NM001012406
*Pdh*	F: ATGAAGAGGGAGGGTGGGAGTTTC; R: GGCGATAGATGGAGTTCCTGTTGTG	XM_003360244.4
*Pfk*	F: GGCTTTGAGGCTTACACAGG; R: GGATGACCACAAACGGGATG	XM_021091211.1
*Pk*	F: TCGATGAGATCCTGGAAGCC; R: TCTTCTGAGCCAGGAAGACC	XM_021099125.1
*Sdh*	F: TGCGAACGGAACCATAAGGACATC; R: GTGCTCCTGGAACGGCTTCTTC	XM_021076931.1
*Tfam*	F: GCTCCTCCTCCTTCGTCGTAGTC; R: GCACCCGTAGACAAAGCACTGAC	NM_001130211.1
**For mice**		
*Bcat2*	F: GCGTCATCTTGCCTGGAGTAGTTC; R: TTCCTTCATAGTGACCTTGCGTTCTG	
*Bckdh*	F: TGGTGGGATGAGGAACAGGAGAAG; R: TACACATCGGAGAAGAGGAGGCTTG	
*Bckdk*	F: AAGGGAGTAGGAGAAGCAGGAAAGG; GCGTCAAGTGAGGGAACTGGTTAC	
*CoxVa*	F: TTGATGCCTGGGAATTGCGTAAAG; R: AACAACCTCCAAGATGCGAACAG	
*C* *o* *XVIIb*	F: TTTCAGGACGCTTTGCAAGG; R: TGCTTCGAACTTGGAGACGG	
*mCpt-1b*	F: ACTCTTGGAAGAAGTTCA; R: AGTATCTTTGACAGCTGGGAC	
*Cyt* *C*	F: CCAGTGCCACACTGTGGAAAA; R: TCTCCCCAGGTGATGCCTTT	
*Gapdh*	F: CATCACTGCCACCCAGAAGACTG; R: ATGCCAGTGAGCTTCCCGTTCAG	
*L* *cad*	F: GTAGCTTATGAATGTGTGCACTC; R: GTCTTGCGATCAGCTCTTTCATTA	
*Ldh*	F: GGAAGGAGGTTCACAAGCAG; R: TCACAACATCCGAGATTCCA	
*M* *cad*	F: GATCGCAATGGGTGCTTTTGATAGA A; R: AGCTGATTGGCAATGTCTCCAGCAAA	
*MyHCI*	F: CCAAGGGCCTGAATGAGGAG; R: GCAAAGGCTCCAGGTCTGAG	
*MyHCIIa*	F: AAGCGAAGAGTAAGGCTGTC; R: GTGATTGCTTGCAAAGGAAC	
*MyHCIIb*	F: ACAAGCTGCGGGTGAAGAGC; R: CAGGACAGTGACAAAGAACG	
*MyHCIIx*	F: CCAAGTGCAGGAAAGTGACC; R: AGGAAGAGACTGACGAGCTC	
*MyoD*	F: GAGGATCCGATGGAGCTTCTATCG; R: CGGATCCTCTCAAAGCACCTGATA	
*MyoG*	F: TTTGCAGTGGATCTTGGGAACCTTC; R: GTCAGACGGCAGCTTTACAAACAAC	
*Pdh*	F: GAAGGCCCTGCATTCAACTTC; R: ATAGGGACATCAGCACCAGTGA	
*Pfk*	F: CAGTCAGTGCCAACATAACCAA; R: CGGGATGCAGAGCTCATCA	
*Pk*	F: TGCCGTGCTGAATGCCTGGG; R: CGCCACCCGGTCAGCACAAT	
*Sdh*	F: GAAAGGCGGGCAGGCTCATC; R: CACCACGGCACTCCCCATTTT	
*Tfam*	F: GCTCTACACGCCCCTGGTTTCTGG; R: TCGCTGTAGTGCCTGCTGCTCCTG	

**Table 2:** Alpha diversity indices of fecal bacteria of pigs and mice.

Indices	Results							
	Pigs				Mice			
	EH	LY	SEM	*p*	CG	AG	SEM	*p*
Chao 1	842.6 ^a^	765.3 ^b^	25.81	0.200	497.7 ^a^	591.7 ^b^	12.77	0.007
Simpson	0.02 ^a^	0.04 ^b^	0.004	0.004	0.07 ^a^	0.03 ^b^	0.01	0.016
Shannon	4.96 ^a^	4.58 ^b^	0.07	0.004	3.89 ^a^	4.41 ^b^	0.08	0.023
Good’s Coverage	>99%	>99%			>99%	>99%		

The data are expressed as the means and SEM. In the same row, values with different superscripts are significantly different (*p* < 0.05).

## Data Availability

The fecal 16S rRNA gene sequences of pigs and mice were deposited into the NCBI Sequence Read Archive (SRA) under the accession number PRJNA723974 (https://www.ncbi.nlm.nih.gov/bioproject/PRJNA723974) (accessed on 23 April 2021).
